# Biomechanical feasibility of semi-rigid stabilization and semi-rigid lumbar interbody fusion: a finite element study

**DOI:** 10.1186/s12891-021-04958-3

**Published:** 2022-01-03

**Authors:** Chia-En Wong, Hsuan-Teh Hu, Li-Hsing Kao, Che-Jung Liu, Ke-Chuan Chen, Kuo-Yuan Huang

**Affiliations:** 1 Section of Neurosurgery, Department of Surgery, National Cheng Kung University Hospital, College of Medicine, National Cheng Kung University, Tainan, Taiwan; 2 Department of Civil Engineering, National Cheng Kung University, Tainan, Taiwan; 3 Department of Civil and Disaster Prevention Engineering, National United University, Miaoli, Taiwan; 4 Department of Orthopedics, National Cheng Kung University Hospital, College of Medicine, National Cheng Kung University, Tainan, Taiwan

**Keywords:** Posterior lumbar interbody fusion (PLIF), Semi rigid stabilization, Semi-rigid interspinous spacer, Adjacent degeneration, Finite element

## Abstract

**Background:**

Semi-rigid lumbar fusion offers a compromise between pedicle screw-based rigid fixation and non-instrumented lumbar fusion. However, the use of semi-rigid interspinous stabilization (SIS) with interspinous spacer and ligamentoplasty and semi-rigid posterior instrumentation (SPI) to assist interbody cage as fusion constructs remained controversial. The purpose of this study is to investigate the biomechanical properties of semi-rigidly stabilized lumbar fusion using SIS or SPI and their effect on adjacent levels using finite element (FE) method.

**Method:**

Eight FE models were constructed to simulate the lumbosacral spine. In the non-fusion constructs, semi-rigid stabilization with (i) semi-rigid interspinous spacer and artificial ligaments (PD-SIS), and (ii) PI with semi-rigid rods were simulated (PD + SPI). For fusion constructs, the spinal models were implanted with (iii) PEEK cage only (Cage), (iv) PEEK cage and SIS (Cage+SIS), (v) PEEK cage and SPI (Cage+SPI), (vi) PEEK cage and rigid PI (Cage+PI).

**Result:**

The comparison of flexion-extension range of motion (ROM) in the operated level showed the difference between Cage+SIS, Cage+SPI, and Cage+PI was less than 0.05 degree. In axial rotation, ROM of Cage+SIS were greater than Cage+PI by 0.81 degree. In the infrajacent level, while Cage+PI increased the ROM by 24.1, 27,7, 25.9, and 10.3% and Cage+SPI increased the ROM by 26.1, 30.0, 27.1, and 10.8% in flexion, extension, lateral bending and axial rotation respectively, Cage+SIS only increased the ROM by 3.6, 2.8, and 11.2% in flexion, extension, and lateral bending and reduced the ROM by 1.5% in axial rotation. The comparison of the von Mises stress showed that SIS reduced the adjacent IVD stress by 9.0%. The simulation of the strain energy showed a difference between constructs less than 7.9%, but all constructs increased the strain energy in the infradjacent level.

**Conclusion:**

FE simulation showed semi-rigid fusion constructs including Cage+SIS and Cage+SPI can provide sufficient stabilization and flexion-extension ROM reduction at the fusion level. In addition, SIS-assisted fusion resulted in less hypermobility and less von Mises stress in the adjacent levels. However, SIS-assisted fusion had a disadvantage of less ROM reduction in lateral bending and axial rotation. Further clinical studies are warranted to investigate the clinical efficacy and safety of semi-rigid fusions.

## Background

Posterior lumbar interbody fusion (PLIF) has been the gold standard procedure for patients with lumbar disc degeneration unresponsive to conservative treatments with excellent clinical outcomes [1–3]. In addition to the placement of interbody implants, rigid fixation using posterior instrumentation (PI) with pedicle screw-based construct was commonly performed in combination with PLIF to provide immediate stabilization of the spine and promote bone fusion [4, 5]. However, rigid fixation markedly alters the biomechanics of the spine, often causing decreased motion at the operated level and a subsequent compensatory increase of motion and intradiscal pressure at the adjacent levels, which further contribute to accelerated adjacent segment degeneration (ASD) [6–8]. As a result, non-instrumented lumbar fusion has always been a well-established alternative method to instrumented PLIF [9, 10]. In single-level lumbar disease, previous studies have shown that non-instrumented lumbar fusion provided comparable results in clinical satisfaction [5, 11].

More versatile implants were developed to assist and promote lumbar fusion [12–16]. Semi-rigid fusion strategies were proposed as a compromise between pedicle screw based rigid fixation and non-instrumented lumbar fusion [17, 18]. By allowing a small-ranged, limited spinal motion, the use of semi-rigid stabilization strategies has been advocated to achieve a more physiologic bone fusion and reduce the undesirable effects following rigid fixation, such as stress shielding of the interbody space, screw loosening at the bone interface, construct fatigue, fractures of the pedicle, and ASD [19, 20]. Semi-rigid stabilization implants included semi-rigid PI (SPI) and semi-rigid interspinous spacer (SIS) with ligamentoplasty [8]. Previous studies showed SPI provided comparable stability to rigid fixation while maintaining limited motions [21, 22]. Although, the use of SPI with lumbar interbody cage were evaluated, the impact of the constructs on the superior and inferior adjacent levels remained uninvestigated [21, 23].

Originally designed as an implant for non-fusion lumbar surgery, the off-label use of SIS with ligamentoplasty in combination with interbody cage as a fusion construct was discussed but remained controversial [24]. Biomechanical studies showed that the combined use of SIS and ligamentoplasty could provide effective limitation in spinal motion[25–27]. The results by Tsai et al. revealed the SIS device Coflex allows limited motion that is significantly less than the motion found in the destabilized spine, and the allowed motion is not significantly different from the intact specimens[27]. The in vitro and FE study by Lafage et al. indicated a decrease of disc stresses and increase of loads transmitted to the spinous processes following SIS[28]. Gonzalez-Blohm et al. evaluated the biomechanics of the ASPEN SIS device in PLIF constructs and they suggested that the SIS device and PI performed equivalently in flexion-extension, but the pedicle screws construct was more resistant to lateral bending[29]. Moreover, compared to pedicle screw-based constructs, SIS and ligamentoplasty have several advantages in terms of smaller incision, shorter operative time, less intraoperative blood loss, and less muscle dissection [30]. The preservation of the paravertebral musculature integrity results in better soft tissue support and benefits patients’ post-operative recovery [31].

Despite the potential advantages of semi-rigid implants, given the paucity of clinical evidence and lack of biomechanical evaluations, the use of semi-rigid fusion was not well-established. Although previous efforts were made to investigate lumbar fusion with SPI, critical parameters relevant to the development of ASD such as the stress and strain energy on both superior and inferior adjacent discs remained poorly-evaluated [21, 23]. Moreover, the effect of these constructs on adjacent levels with preexisting degeneration was also uninvestigated.

In the present study, we established a FE model of the lumbosacral spine and simulated Pfirrman grade IV disc degeneration in L4-5[32]. Eight models were simulated, including the intact model, posterior decompression surgery (PD), PD with SIS stabilization and ligamentoplasty (PD + SIS), PD with SPI (PD + SPI), non-instrumented fusion with interbody cage implantation (Cage), semi-rigid lumbar fusion with cage implantation, SIS stabilization, and ligamentoplasty (Cage+SIS), semi-rigid lumbar fusion with cage and SPI (Cage+SPI) and instrumented PLIF with cage and pedicle screw instrumentation (Cage+PI). This study was aimed to evaluate the biomechanical property of semi-rigid stabilization and semi-rigid lumbar fusion. The range of motion (ROM) of the operated level were compared. We investigated the impact of different constructs on the adjacent levels simulated with preexisting degeneration. Critical parameters relevant to the development of ASD including ROM, intervertebral discs (IVD) stress, and strain energy were compared.

## Method

### Generation of lumbosacral FE model

**Fig. 1:**
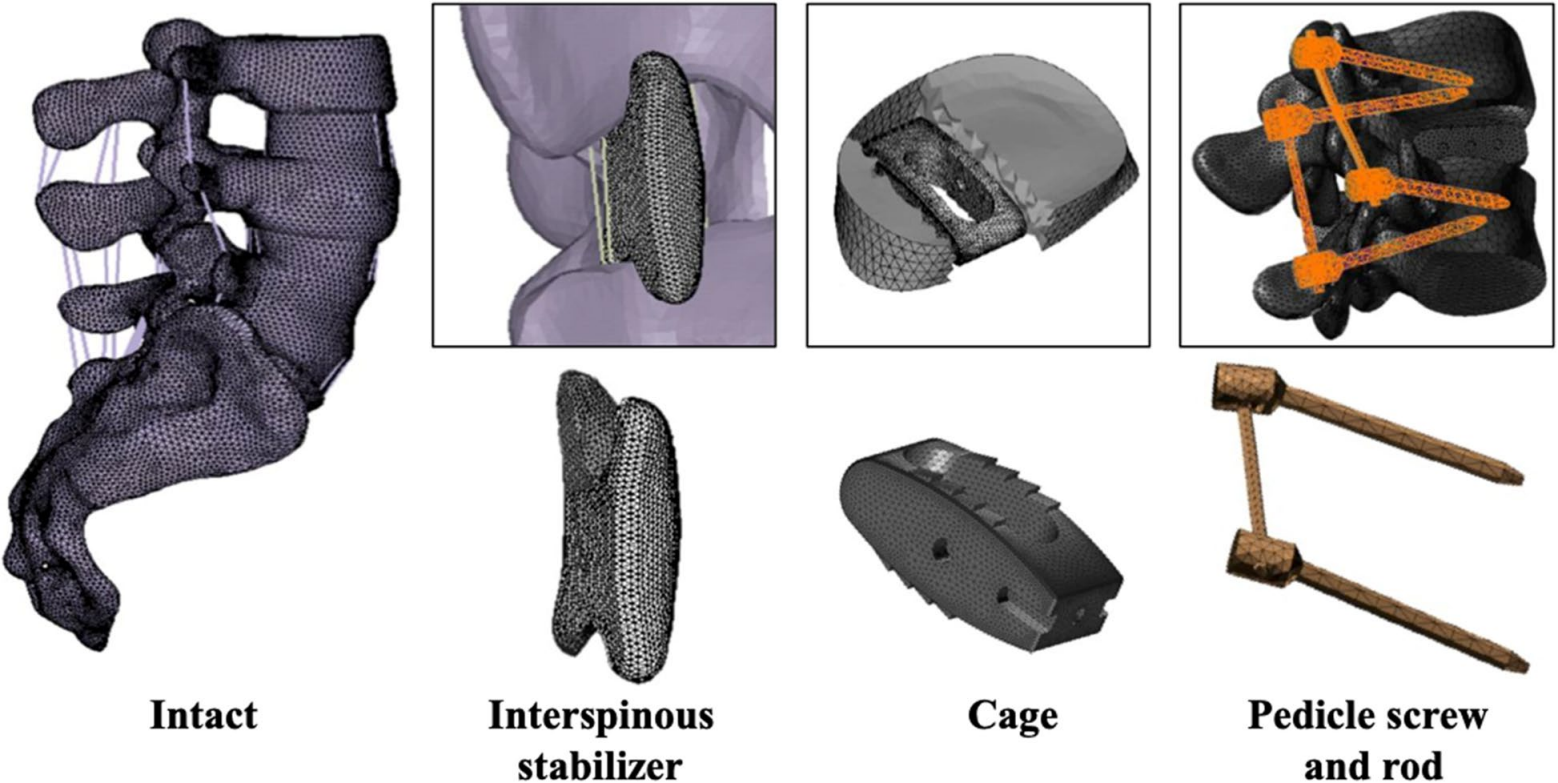
Finite element model of L3-Sacrum segments and surgical implants. The present finite element model of the intact spine (left), interspinous spacer (middle-left), interbody cage (middle-right) and pedicle screws and rods (right)

A three-dimensional FE model of the lumbosacral spine from the third lumbar vertebra to the sacrum (L3-S5) was developed from the axial computed tomography (CT) images at 1-mm thickness (512 × 512) resolution, 16-bit, and a pixel size of 0.3516 mm × 0.3516 mm, obtained from a resin spine model casted from a cadaveric spine. The CT images were imported into 3D-DOCTOR (Able Software Corp.) to construct the geometric structure of L3-Sacrum. The mesh was then prepared using Patran (MSC Software) and the FE model was imported into Abaqus 6.12 (Simulia Inc) to solve (Fig. 1). This study adopted linear and isotropic material properties for cortical bone, cancellous bone, posterior bony elements, endplate, annulus fiber layers, annulus ground substance, and nucleus pulposus (Table 1).

**Table 1:** Material properties of the lumbosacral FE model and spinal implants

Component	Young’s Modulus (MPa)	Poisson^’^s Ratio
Annulus Fiber		
Inner Laminate: Inner Layer	360	0.30
Inner Laminate: Middle Layer	385	0.30
Inner Laminate: Outer Layer	420	0.30
Outer Laminate: Inner Layer	440	0.30
Outer Laminate: Middle Layer	495	0.30
Outer Laminate: Outer Layer	550	0.30
Annulus Ground Substance	4.2	0.45
Nucleus Pulposus	1	0.49
Cancellous Bone	100	0.20
Cortical Bone	12,000	0.30
Posterior Bony Elements	3500	0.25
Endplate	12,000	0.30
ALL/PLL/LF/ISL/SSL	20/20/20/10/10	0.25
DIAM core	20	0.45
DIAM Truss	5000	0.20
Graft Bone	100	0.20
PEEK	3600	0.25
Titanium screw/rod	110,000	0.30

A vertebra was composed of a cortical bone shell (thickness, 0.35 mm), cancellous bone, endplates (thickness, 0.5 mm), and posterior elements. A closed surface of cortical bone and endplates was assigned to 3-node S3R shell elements. Cancellous bone was assigned to 4-node C3D4 tetrahedral elements. The irregular posterior elements including the facet joints were modeled using C3D4 elements according to the original geometry. The contact of the facets was simulated with three-dimensional surface-to-surface contact with friction and a finite sliding interaction was defined with the friction characteristic modeled with classic isotropic Coulomb friction model and a friction coefficient of 0.1 [33–35].

The FE model of the IVD consisted of annulus fiber layers, annulus ground substance and nucleus pulposus. The height of the discs at L3-4, L4-5, L5-S1 levels were 9.802, 7.65, and 10.34 mm, respectively. The model of the annulus fibrosus was created as ring-shaped by generating an outer border and an inner border using outer annulus fiber and inner annulus fiber, respectively. The superior and the inferior border of the annulus fibrosus were the adjacent endplates. The annulus fibers were modeled with six layers of shell elements with the thickness of each layer of 1.5 mm. Annulus ground substance were defined between the two annulus fibers and the adjacent endplates and was modeled by C3D4 tetrahedral elements. Nucleus pulposus were defined by the inner annulus fiber and adjacent endplates and was modeled by C3D4elements. In the present study, Pfirrmann grade IV moderate degeneration in L4-5 and Pfirrmann grade III low grade degeneration in the adjacent L3-4 and L5-S1 IVDs were simulated. In Pfirrmann grade III degeneration, the disc height was preserved [32, 36]. However, the annulus degenerated and previous study reported a decrease of annulus fibrosus elastic modulus in the mildly to moderately degenerated IVD [37, 38]. With this in mind, the degenerated IVD was simulated by modifying the material properties of the annulus fibrosus with the elastic moduli of the annulus fiber layers reduced by 80% in L4-5 to simulate moderately degenerated IVD and 20% in L3-4, L5-S1 to simulate mildly degenerated adjacent levels [39].

Ligamentous complex including anterior longitudinal ligaments (ALL), posterior longitudinal ligaments (PLL), ligamentum flavum (LF), interspinous ligaments (ISL) and supraspinous ligaments (SSL) were modeled using hyperelastic, tension-only, Truss elements (T3D2). The properties of the ligaments were adopted from Goel et al. [40] and given in Table 2. The element types and number of elements used in the components of the spine are listed in Table 3.

**Table 2:** Properties of the ligaments in the present study

Ligament	ALL	PLL	LF	ISL	SSL
Elastic modulus (small strain)(MPa)	7.8	10	15	10	8
Transition strain (%)	12	11	6.2	14	20
Elastic modulus (large strain)(MPa)	20	20	19.5	11.6	15
Cross sectional area (mm2)	52.8	16	66.8	26	23

**Table 3:** Element type and number of element in the intact lumbosacral FE model

Component	Element type	No. of Elements				
		L3	L4	L5	Sacrum	
Cortical Bone	S3R	1334	1913	2027	30,212	
Cancellous Bone	C3D4	18,166	20,129	22,756	131,828	
Endplate	S3R	1833	2013	2193	1615	
Posterior Bony Elements	C3D4	26,695	27,739	25,313		
		**L3-L4**	**L4-L5**	**L5-S1**		
Nucleus Pulposus	C3D4	4942	3998	5670		
Annulus Ground Substance	C3D4	8262	4619	7063		
Annulus Fiber	STRI3	1963	1143	1350		
**Ligament**		ALL	PLL	LF	ISL	SSL
**No. of Elements**	T3D2	12	12	12	6	6

### Generation of the implant models

The implant model for the SIS was generated according to the DIAM interspinous (Medtronic) implant. The primary dimensions (width, length, heights) were 14 mm, 28 mm, and 12 mm respectively, and the interspinous distance of the implant body in between the two wings was 12 mm. The interspinous implant composed of a silicon core covered inside a polyester mesh, which connects to a pair of polyester mesh tethers that ties the spacer on to the adjacent spinous processes for ligamentoplasty. The three-dimensional structure of the interspinous spacer was created in software Patran (MSC Software Corp.) and mesh structures were prepared using software Hypermesh 11.0 (Altair Technologies Inc) (Fig. 1). The implant body composed of C3D4 solid elements, and the tethers were assigned to tension-only Truss element. The contact surface between the spinous process and the interspinous spacer was assumed with a coefficient of friction of 0.5.

For interbody fusion, the model for lumbar interbody cage was developed from the CAPSTONE® PEEK Spinal System (medtronic) using Patran software (MSC Software Corp.). Mesh structures were prepared and assigned to C3D4 solid elements using software Hypermesh 11.0(Altair Technologies Inc). The cage composed of polyetheretherketone (PEEK) and the primary dimensions (width, length, heights) were 14 mm, 26 mm and 10 mm, respectively (Fig. 1). The primary dimensions (diameter, length) of the pedicle screws for lumbar posterior instrumentation were 5.5 mm × 45 mm and 6.5 mm × 45 mm, respectively. The diameter of the rods was 6.0 mm (Fig. 1). The pedicle screws and the rigid rods were composed of titanium. The semi-rigid rods were composed of PEEK. The material properties and element types assigned to the implants were shown in Table 1.

### Simulation of surgeries

**Fig. 2:**
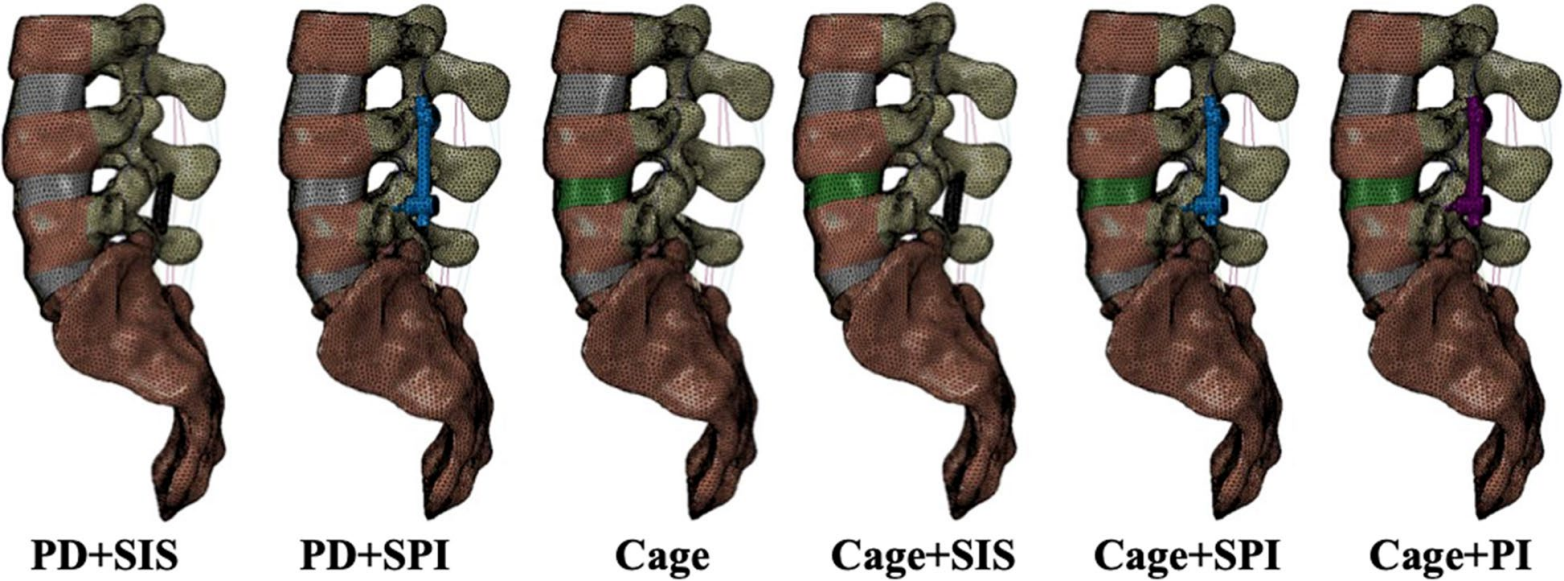
Different L4/5 surgical constructs. Surgical constructs simulated in this study including PD + SIS, PD + PSI, Cage, Cage+SIS, Cage+SPI, and Cage+PI. Interbody fusion with cage implantation was indicated in green. Semi-rigid and rigid instrumentation were indicated in blue and purple, respectively

Eight models were simulated, including the intact model, posterior decompression surgery (PD), PD with SIS stabilization and ligamentoplasty (PD + SIS), PD with SPI (PD + SPI), non-instrumented fusion with interbody cage implantation (Cage), semi-rigid lumbar fusion with cage implantation, SIS stabilization, and ligamentoplasty (Cage+SIS), semi-rigid lumbar fusion with cage and SPI (Cage+SPI) and instrumented PLIF with cage and pedicle screw instrumentation (Cage+PI) (Fig. 2).

For L4-5 posterior decompression, a unilateral interlaminar decompression with medial facetectomy was simulated by removing the inferior half of the L4 laminar, the superior one-fourth of the L5 laminar and the medial twenty-percent of the L4-5 articular facets. Interbody cage fusion over L4-5 was performed with cage placement after total removal of the nucleus and partial removal of the PLL and posterior annulus.

### Loading and boundary conditions

The boundary conditions imposed in this study for all models were set with the nodes on the sacroiliac joint surface constrained in all directions. The facet interaction and the spinous process-interspinous spacer interaction were defined as finite sliding, surface-to-surface contact model with classical Coulomb friction. The endplate-cage boundaries were defined as tie-constraint.

A preload of 150 N was applied evenly on the superior endplate of the L3 vertebral body using follower load technique to stimulate the axial loading of the lumbosacral spine. For simulations of lumbosacral motions including flexion, extension, lateral bending, and axial rotation, a 3 N-m moment was applied evenly on the L3-L5 segments, with a moment of 1 N-m on each segment. The rationale behind the evenly distributed moment application is that the primary muscles producing lumbar spine motions such as the psoas muscle (flexion), erector spinae and semispinalis (extension), quadratus lumborum and external/internal obliques (lateral bending), semispinalis, and deep posterior paraspinal muscles (rotation) all had multiple attachments on multiple lumbar segments. As a result, an evenly distributed moment could represent a more physiological condition.

### Convergence test

We used the intact model for convergence test and measured the displacement of a reference point on the center of the L3 superior endplate under a 150 N preload. Four different amounts: 516783, 509,525, 354,824, and 272,588 elements were compared for their corresponding displacements using. 516,783 elements as reference. The final FE model consisted of 354,824 elements connected through 69,134 nodes. The relative displacement error was less than 3.75% and the element size ranged from 1.0 to 2.0 mm.

## Results

### Model validation

**Fig. 3:**
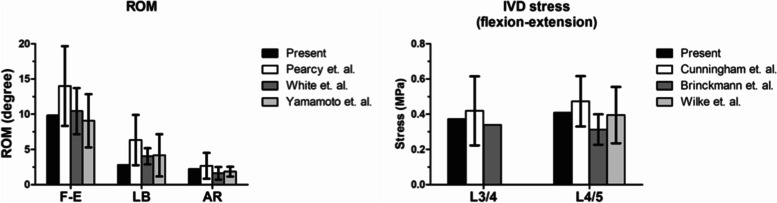
Validation of the present finite element model. Comparisons between the ROM and IVD stress of the present intact model with the literature (presented in mean and standard deviation)

For the validation of the FE model, we compared the simulated ROM and IVD stress of the intact model with those reported in the literature (Fig. 3). First, the ROMs of the present intact FE model of the lumbosacral spine were compared with the in vivo measurements and in vitro experiments conducted by Pearcy et al., White et al., and Yamamoto et al. [41–44]. The average segmental ROMs of the present intact model were as follows: flexion-extension, 8.85; lateral bending, 2.31; and axial rotation, 2.22 (Degrees). The ROMs were in good agreements with the literature.

Second, the model was validated with the in vitro measurements of lumbar intradiscal pressure conducted by Cunningham et al., Brinckmann et al. and Wilke et al. [45–47]. In the previous in vitro experiments, the average stress in the IVD under sagittal flexion-extension ranged from 0.22 Mpa to 0.61 MPa in L3-4 and 0.23 MPa to 0.62 MPa in L45. The IVD stress under flexion-extension in the present model were 0.37 Mpa in L3-4 and 0.41 MPa in L4-5, which were compatible with the in vitro results (Fig. 3).

### ROM at the operated level

**Fig. 4:**
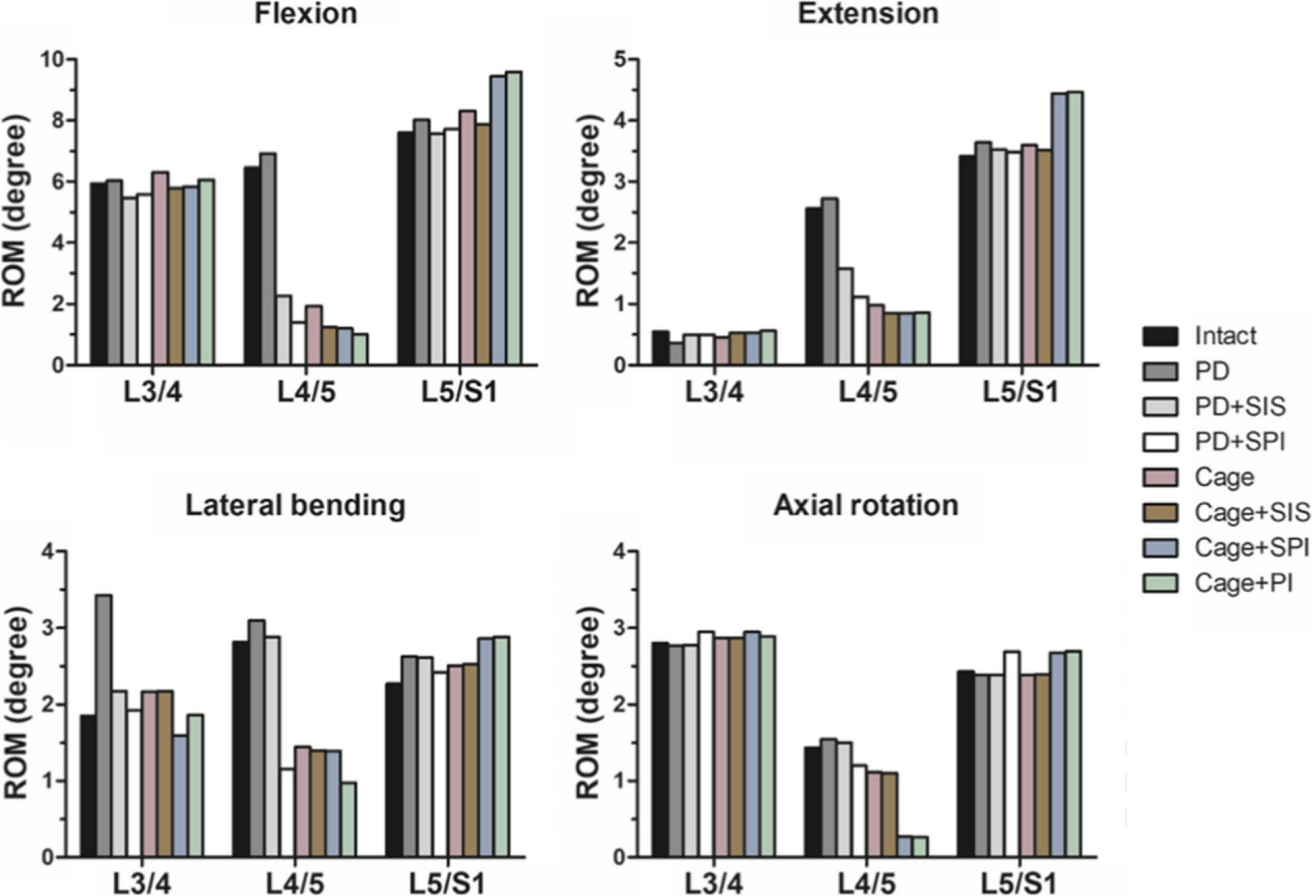
The segmental ROM of the intact and surgical models. The simulated segmental ROM in flexion, extension, lateral bending, and axial rotation

The ROM of each construct under flexion, extension, lateral bending and axial rotation were shown in Fig. 4. Compared to the intact model, PD resulted in hypermobility at the operated level under all motions, which is accompanied by a marked increase in ROM in the suprajacent level (L3-4) during lateral bending and a mild increase in ROM in the infrajacent level (L5-S1) during flexion, extension and lateral bending. The application of semi-rigid stabilization with SIS after PD (PD + SIS) decreased the ROM of the decompressed level. Compared to PD + SIS, PD + SPI had more ROM reduction at the operated level in all motion, especially in lateral bending.

Comparison between the constructs intended to achieve fusion including Cage, Cage+SIS, Cage+SPI and Cage+PI revealed that all fusion constructs reduced the ROM at the operaed level. In flexion, Cage+PI had the most ROM reduction, while Cage only had the least ROM reduction. The difference between Cage+SIS and Cage+SPI was less than 0.03 degree. In extension, while Cage had the least ROM reduction, the differences between Cage+SIS, Cage+SPI, and Cage+PI were less than 0.05 degree. In lateral bending, Cage+PI had better ROM reduction than other three constructs. In axial rotation, pedicle screw-based constructs (Cage+SPI and Cage+PI) had significant more ROM reduction compared to Cage and Cage+SIS. The difference between Cage+SPI and Cage+PI was less than 0.02 degree in axial rotation. In summary, the ROM reduction of semi-rigid fusion constructed were better than non-instrumented fusion. Comparing Cage+SIS and Cage+SPI instrumented fusion (Cage+PI), the result showed that the three fusion strategies had comparable performance in ROM reduction in the operated level in flexion and extension, but Cage+PI had a better ROM reduction in lateral bending. The major difference of Cage+SIS and Cage+SPI was observed in axial rotation with Cage+SPI had better ROM reduction than Cage+SIS. The values of the ROM were shown in Table 4.

**Table 4:** ROM of the intact and surgical models

Flexion	Intact	PD	PD + SIS	PD + SPI	Cage	Cage+SIS	Cage+SPI	Cage+PI
L3/4	5.94	6.03	5.47	5.58	6.30	5.78	5.83	6.05
L4/5	6.45	6.93	2.27	1.40	1.93	1.24	1.21	1.01
L5/S1	7.60	8.02	7.57	7.72	8.31	7.88	9.45	9.59
Extension	Intact	PD	PD + SIS	PD + SPI	Cage	Cage+SIS	Cage+SPI	Cage+PI
L3/4	0.55	0.36	0.50	0.51	0.45	0.53	0.53	0.56
L4/5	2.56	2.72	1.58	1.12	0.98	0.85	0.86	0.88
L5/S1	3.42	3.64	3.53	3.49	3.6	3.52	4.45	4.46
Lateral bending	Intact	PD	PD + SIS	PD + SPI	Cage	Cage+SIS	Cage+SPI	Cage+PI
L3/4	1.85	3.43	2.18	1.92	2.17	2.18	1.60	1.86
L4/5	2.82	3.11	2.88	1.15	1.45	1.40	1.39	0.98
L5/S1	2.27	2.63	2.62	2.42	2.51	2.53	2.86	2.89
Axial rotation	Intact	PD	PD + SIS	PD + SPI	Cage	Cage+SIS	Cage+SPI	Cage+PI
L3/4	2.80	2.77	2.78	2.95	2.87	2.87	2.95	2.89
L4/5	1.44	1.55	1.51	1.23	1.15	1.12	0.27	0.26
L5/S1	2.43	2.39	2.39	2.69	2.39	2.42	2.68	2.69

### ROM at the adjacent levels

Semi-rigid stabilization with SIS or SPI after PD resulted ROMs similar to that of the intact spine. Moreover, the significant increase in lateral bending ROM after PD was alleviated in PD + SIS and PD + SPI (Fig. 4). Comparison between the fusion models showed that all four fusion constructs had comparable ROM to the intact spine in the suprajacent level(L3-4) in flexion, extension, and axial rotation. In lateral bending, while Cage+SPI reduced the ROM at the suprajacent level by 13%, a 17, 18, and 9% increased motion in lateral bending in the suprajacent level was noted for Cage, Cage+SIS, and Cage+PI, respectively.

For the infrajacent level, pedicle screw-based constructs resulted in significant hypermobility of all motions with 24.1, 27,7, 25.9, and 10.3% ROM increase for Cage+SPI and 26.1, 30.0, 27.1, and 10.8% ROM increase for Cage+PI in flexion, extension, lateral bending and axial rotation respectively. In contrast, Cage and Cage+SIS only increased the motion at the infrajacent level by 9.3 and 3.6% in flexion, 5.3 and 2.8% in extension, 10.5 and 11.2% in lateral bending, respectively. Moreover, Cage and Cage+SIS reduced the ROM by 1.8 and 1.5% in axial rotation compared to the intact model. The values of the ROM were shown in Table 4.

### The von Mises stress and strain energy at the operated level

**Fig. 5:**
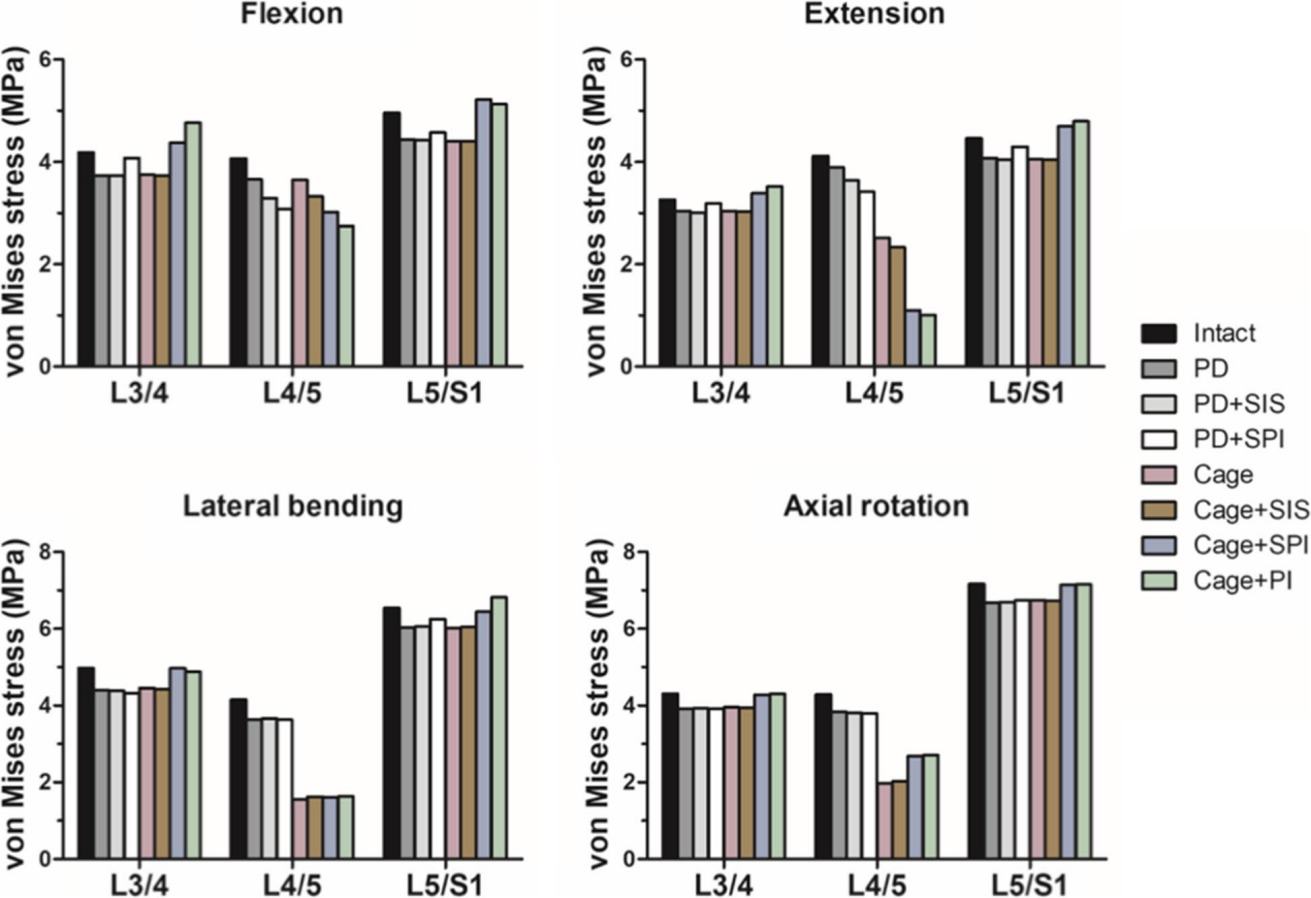
The maximum von Mises stress in the intact and surgical models. The maximum von Mises stress in the operated level and the adjacent levels in flexion, extension, lateral bending, and axial rotation

The maximal von Mises stress of the intervertebral disc or the interbody cage at the operated level (L4-5) of each construct were shown in Fig. 5. All constructs decreased the maximal von Mises stress at the operated level (L4-5) in all motions. In flexion, Cage, Cage+SIS, Cage+SPI, and Cage+PI reduced the stress at the fusion level by 10.3.0, 18.2, 25.8, and 32.4% compared to the intact model, respectively. In extension, Cage, Cage+SIS, Cage+SPI, and Cage+PI reduced the stress by 38.7, 43.4, 73.3, and 75.7%, respectively. In lateral bending, the differences of the stress in the implanted cage were less than 4.3%. In axial rotation, higherstress of the interbody cage was found in Cage+SPI and Cage+PI while Cage and Cage+SIS had less stress.

**Fig. 6:**
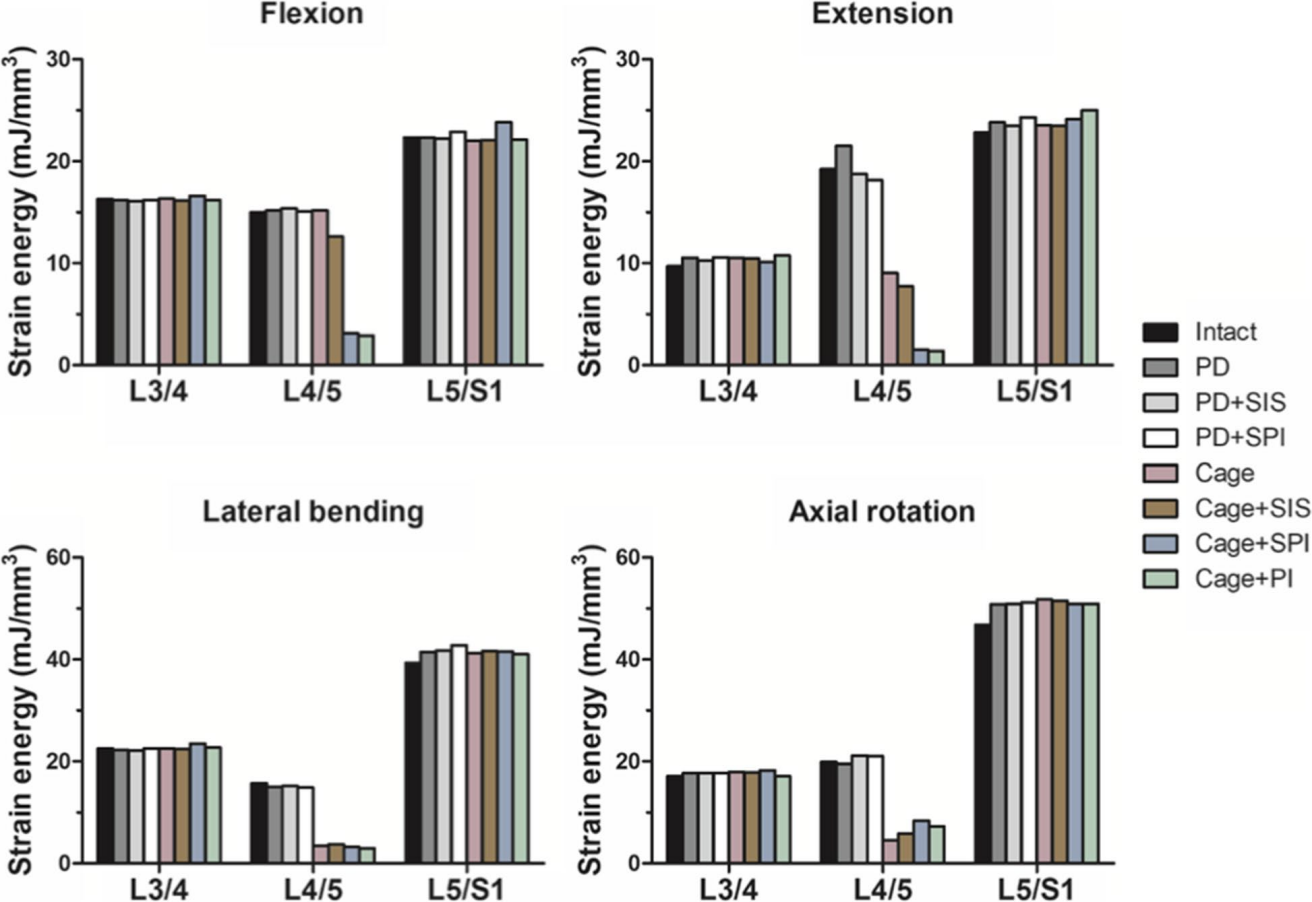
The maximum strain energy in the intact and surgical models. The maximum strain energy in the operated level and the adjacent levels in flexion, extension, lateral bending, and axial rotation

The strain energy in the L4-5 intervertebral disc was comparable among non-fusion constructs in flexion, lateral bending, and axial rotation. In extension, PD resulted in an increased strain energy at the operated level, which can be alleviated by semi-rigid stabilization. (Fig. 6). All fusion constructs decreased the strain energy of the operated level in extension, lateral bending, and axial rotation. The main difference in strain energy reduction was noted in flexion and extension. In flexion, Cage+SPI and Cage+PI reduced the L4/5 strain energy markedly by 79.1 and 80.8%, respectively, but Cage+SIS only had 15.9% decrease. Moreover, fusion with interbody cage only (Cage) was unable to reduce the L4/5strain energy.

### The von Mises stress and strain energy at the adjacent intervertebral disc

Compared to the intact model, all non-fusion constructs had decreased IVD stress at both adjacent levels in all motions. For fusion constructs, Cage+SPI and Cage+PI resulted in increased von Mises stress at both adjacent levels in all motions compared to Cage and Cage+SIS. In contrast, Cage, and Cage+SIS had decreased von Mises stress compared to the intact model in all motions. The average percentage of decrease in the von Mises stress at the adjacent levels compared to the intact model was 8.7% and 9.0% for Cage and Cage+SIS, respectively.

In terms of the strain energy of the adjacent IVDs, the difference of the strain energy at the suprajacent level among all models was less than 7.9% (Fig. 6). In contrast, compared to the intact model, the strain energy at the infrajacent level under extension, lateral bending, and axial rotation was increased in allsurgical constructs, but the percentages of increase were all less than 9%.

## Discussion

To investigate the impact of the semi-rigid fusion constructs on the fusion and the adjacent levels, the present study utilized FE model to simulate semi-rigid non-fusion and fusion constructs involving SIS and SPI and compared them to rigid PI constructs. We showed Cage+SIS, and Cage+SPI provided similar stabilization in flexion-extension and lateral bending of the fusion level compared to Cage+PI but Cage+SIS was less resistant to axial rotation. Our work revealed Cage+SIS had advantages in less increase of infradjacent ROM and reduced adjacent IVD stresses compared to SPI and PI. The difference in strain energy between the constructs was less than 7.9%, but all constructs increased the strain energy in the infradjacent level.

In the current FE model, we simulated preexisting disc degeneration on both adjacent levels and compared critical parameters relevant to the development of ASD such as ROM, IVD stress, and strain energy. Preexisting disc degeneration is a major risk factor for ASD[48]. Since we aimed to evaluate the impact of the constructs on the adjacent levels, the assumption of preexisting degeneration is more clinically relevant compared to simulation with intact discs. The present model was validated with previously published in vitro experiments. The simulated ROM and IVD stress were compatible with the cadaveric results in the literature [38–44]. Since lumbosacral disc degeneration is most prevalent at L4/5 followed by L5-S1 and L3-4 [49, 50], we chose L4/5 as the operated level and aimed to optimize the lumbar constructs in order to prevent ASD especially at the adjacent level after lumbar interbody fusion.

Pseudarthrosis is a well-known complication of lumbar fusion. To prevent pseudarthrosis, sufficient stabilization to minimize the motion at the fusion level is of paramount importance[51]. Previous study evaluated SIS device in PLIF constructs and suggested that the SIS device and PI performed equivalently in flexion-extension, but the pedicle screws construct was more resistant to lateral bending[29]. Our simulation showed that semi-rigid lumbar fusion Cage+SIS and Cage+SPI had comparable ROM reduction to rigid PI (Cage+PI) at the fusion level under flexion-extension. In lateral bending and axial rotation, only Cage+SPI had comparable ROM reduction to Cage+PI. Together, these results suggested SIS-based lumbar fusion had better stabilization in flexion-extension but was less resistant to lateral bending and rotation. Since excessive motion at the level intended to fuse may hinder bony fusion and was associated non-union [52], further clinical study is needed to investigate whether additional maneuvers such as application of lumbar bracing to limit lateral axial rotation may be helpful to provide support and facilitate fusion after a semi-rigid lumbar fusion with SIS. However, it should be noted that since the primary motion of the lumbar spine is flexion-extension [53], the semi-rigid constructs provided sufficient stabilization in the primary lumbar motion.

Hypermobility in the adjacent levels is a significant risk factor of ASD[54], and preservation of lumbar motion was associated with a lower incidence of lumbar ASD[55]. A construct causing less adjacent hypermobility might help prevent adjacent degeneration and therefore we investigated the adjacent ROM in the semi-rigid constructs. Compared to Cage+SPI and Cage+PI, which caused hypermobility at the infradjacent level, Cage+SIS resulted in a minimal impact in terms of ROM at L5-S1. Our results suggested that semi-rigid lumbar fusion has an advantage in prevention of L5-S1 ASD.

Acute material failure occurs when von Mises stress surpasses the tensile yield stress [56]. For intervertebral discs, it might clinically present as an acutely ruptured annulus with disc herniation compressing the thecal sac or nerve root [57]. Our results revealed that while pedicle screw-based construct (Cage+SPI and Cage+PI) increased the von Mises stress in adjacent discs, Cage+SIS decreased the von Mises stress in adjacent discs. Another clinically relevant parameter is the strain energy at the adjacent intervertebral disc. The strain energy stored in the material during repetitive motion is related to fatigue of the material and this may make the IVD susceptible to chronic disc degeneration and progression of the pre-existing disc degeneration to higher-grade degeneration [58]. An increased strain energy at the infrajacent was found among all surgical constructs in extension, lateral bending, and axial rotation compared to the intact model. Although the percentages of increase were less than 9%, it may imply that all L4/5 surgical constructs may increase the susceptibility of L5S1 to chronic degeneration.

Despite that, the comparisons of von Mises stress and strain energy between Cage+SIS, Cage+SPI and Cage+PI still revealed that Cage+SIS decreased the von Mises stress at adjacent levels and had less increase in strain energy at L5-S1 in flexion-extension. This may suggest Cage+SIS had a pontential advantage in lower risks of ASD. Taken together, our results suggested that Cage+SIS and Cage+SPI can provide sufficient stabilization and ROM reduction to facilitate spinal fusion. In addition, our results showed that semi-rigid lumbar fusion with SIS had less impact on adjacent levels in terms of less hypermobility and less von Mises stress. However, Cage+SIS had a disadvantage of less ROM reduction in lateral bending and axial rotation. Further clinical studies are warranted to investigate whether additional maneuvers such as lumbar bracing should be applied to limit the motions and minimize the disacvantage of Cage+SIS.

The limitations of the present study should be noted. First, the IVD and the bony tissue was modeled using linear isotropic material properties and the anisotropic properties of the materials were neglected. Second, hyperelastic Truss elements were used to simulate the ligaments, which neglected the contact interaction between ligaments and vertebrae but also avoided the unrealistic shearing forces in the ligaments. The simplifications reduced the computation time. Other limitations included that the position and trajectory of the screws and interspinous implants were likely to have variations. Changes in orientation of the implants may alter the biomechanics of the construct but this is very challenging to be incorporated into the simulation since multiple real-world factors such as surgical approach, anatomical variation, and surgeon’s preference all affect the positioning of the implant. Last, perfect surface contact with tie constraints were made between pedicle screws, cage, and bone. This assumption was an advantage over cadaveric experiments since it allowed us to simulate spinal adaptation to bony fusion [59]. Despite these limitations, the main conclusions of the present study are based on comparisons between the surgical models. The above-mentioned model simplifications are applied to all models and likely have minimal effect on the comparative differences.

## Conclusion

Semi-rigid fusion constructs can provide adequate stabilization and flexion-extension ROM reduction at the fusion level similar to rigid fixation in flexion-extension. In addition, SIS-assisted fusion caused less hypermobility and less von Mises stress in the adjacent levels. However, SIS-assisted fusion had a disadvantage of less ROM reduction in lateral bending and axial rotation. Further clinical studies are warranted to investigate whether additional maneuvers such as lumbar bracing should be applied to minimize the disadvantage of SIS-assisted fusion and to evaluate the clinical efficacy and safety of semi-rigid fusions.

## Data Availability

The datasets used and/or analysed during the current study are available from the corresponding author on reasonable request.

## Abbreviations

SIS: Semi-rigid interspinous sstabilization
SPI: Semi-rigid posterior instrumentation
PEEK: Polyetheretherketone
ROM: Range of motion
PLIF: Posterior lumbar interbody fusion
PI: Posterior instrumentation
ASD: Adjacent segment degeneration
IVD: Intervertebral disc
ALL: Anterior longitudinal ligaments
PLL: Posterior longitudinal ligaments
LF: Ligamentum flavum
ISL: Interspinous ligaments
SSL: Supraspinous ligaments
PD: Posterior decompression
